# Interrelationship between insistence on sameness, effortful control and anxiety in adolescents and young adults with autism spectrum disorder (ASD)

**DOI:** 10.1186/s13229-017-0158-4

**Published:** 2017-07-21

**Authors:** Mirko Uljarević, Amanda L. Richdale, David W. Evans, Ru Ying Cai, Susan R. Leekam

**Affiliations:** 1 Olga Tennison Autism Research Centre, School of Psychological Science, La Trobe University, Bundoora, VIC 3086 Australia; 2 Cooperative Research Centre for Living with Autism (Autism CRC), Level 3 Foxtail Building, Long Pocket, The University of Queensland, Brisbane, QLD 4072 Australia; 3 Department of Psychology, Bucknell University, Lewisburg, PA USA; 4 Wales Autism Research Centre, School of Psychology, Cardiff University, Cardiff, Wales UK

**Keywords:** Insistence on sameness, Effortful control, Self-regulation, Anxiety, Autism

## Abstract

**Background:**

Both self-regulation and insistence on sameness (IS) are related to anxiety, which is a common feature of individuals with autism spectrum disorder (ASD). Here, we aimed to characterise the IS-self-regulation-anxiety interrelationship by investigating the potential contribution made by self-regulation, assessed via effortful control (EC), to the IS-anxiety relationship in a sample of adolescents and young adults with ASD.

**Method:**

Seventy-one older adolescents and younger adults with ASD (49 males, 22 females; *M*_age_ = 18.71 years, SD = 2.51, range 14.42–24.81) completed the Adult Repetitive Behaviour Questionnaire-2, Effortful Control Scale of the Adult Temperament Questionnaire and the DSM-5 Dimensional Anxiety Scales.

**Results:**

IS was associated with both EC (*r* = −.39, *p* = .001) and anxiety (*r* = .45, *p* < .001), and anxiety was in turn associated with EC (*r* = −.44, *p* < .001). To characterise the nature of this interrelationship, two mediation analyses were performed using the serial mediation model in PROCESS with 5000 resamples in bootstrapping. There was a significant indirect effect of EC on anxiety, through IS (*b* = −.06; BCa 95% CI [−.13, −.02]), and indirect effect on anxiety through EC (*b* = 1.62; BCa 95% CI [.59, 3.24]) with the mediators accounting for 29.07 and 26.04% of the total effect, respectively.

**Conclusions:**

Our study provides the first exploration of the IS-anxiety-self-regulation link in ASD. The finding that lower levels of self-regulation are related both to anxiety and IS behaviours points to self-regulation as a viable intervention target for both anxiety and IS behaviours.

## Background

Insistence on sameness (IS) refers to complex patterns of rigid, routinised, and ritualistic behaviours that form a class of restrictive and repetitive behaviours (RRBs) and a diagnostic criterion for autism spectrum disorder (ASD) [1]. These behaviours are also part of typical development where they are transient in nature and often serve an adaptive role [2–6]. During the early development of most children, IS behaviours and typical fears and anxiety, such as fear of strangers, as well as cognitively more complex and anticipatory fears tend to follow a similar developmental trajectory [2, 4, 7–10]. Furthermore, normative IS behaviours are likely to occur at times of transition, such as bedtime or mealtime, and are often accompanied by typical fears/anxieties, including fear of the dark or separation anxiety [3]. These observations have led to the suggestion that IS behaviours act as an early form of self-regulation, serving to control or constrain the environment thus limiting unpredictability and reducing ensuing fears and anxiety, eventually reducing as more advanced forms of self-regulation develop [5, 11–14]. In individuals with ASD, IS behaviours tend to be relatively stable over time [15] and negatively impact the functioning of individuals and their families [5, 16]. It is possible that IS behaviours continue to serve a regulatory function in ASD beyond the period when they are adaptive thus negatively affecting development and, in particular, sustaining anxiety. However, this proposal has not been previously tested.

Self-regulation abilities include attentional control, inhibition of dominant and activation of subdominant responses and ability to shift between multiple tasks and/or mental sets. While cognitive and clinical researchers have traditionally assessed these abilities via measures of executive functioning (EF), developmental psychology researchers have considered self-regulation from a temperament perspective and used measures of effortful control (EC). The majority of the research has identified an overlap between EF and EC constructs, at a conceptual level, and also in terms of their developmental trajectory, genetic and neurodevelopmental underpinnings and outcomes/correlates [17, 18]. EF and EC abilities develop gradually, becoming progressively more advanced and complex between early toddlerhood and school years [17–19], a period during which both IS behaviours and fears gradually decrease. Research indicates that individual variation in EF and EC during this normative developmental period relates to the levels of IS behaviours [10, 20] and also predicts later internalizing problems [21–23]. Thus, it seems that the development of more sophisticated and flexible EF and EC forms during childhood results in less reliance on IS behaviours for managing fears [3]. Furthermore, it is reasonable to hypothesise that IS behaviours, if persistent beyond the developmental period when they are adaptive, may negatively impact subsequent development by limiting children’s exposure to situations conducive to developing more elaborate, complex and flexible patterns of EF and EC, as well as other aspects of social, cognitive and emotional development [5, 24].

A large body of research in ASD indicates significant impairments in EF and EC [25–28]. These impairments have been linked to increased internalizing problems, in particular, anxiety [25, 26, 29–31], as well as with RRBs [5]. In turn, anxiety is associated with higher levels of RRBs, most notably, IS behaviours [32–35]. However, the nature of the IS-EF and EC-anxiety interrelationship has not been explored previously in the ASD literature.

Based on the reviewed literature from both typical development and ASD, lower levels of self-regulation, operationalised as either EC or EF, and higher levels of IS behaviours may both be expected to be related directly to higher levels of anxiety. Additionally, IS and self-regulation might also have an indirect effect on anxiety in the following two ways: (1) the association between self-regulation and anxiety is mediated by IS and (2) the association between IS behaviours and anxiety is mediated by self-regulation. Therefore the aim of this study was to provide the first characterisation of the IS-self-regulation-anxiety interrelationship in a cross-sectional sample of older adolescents and young adults with ASD, using EC as a measure of self-regulation.

## Methods

### Participants

Seventy-one adolescents and young adults with ASD (49 males, 22 females; *M*_age_ = 18.71 years, SD = 2.51, range 14.42–24.81) took part in the study. Participants were recruited through various channels including state-based autism organisations, parent support groups, secondary and tertiary education organisations, participant databases and clinicians. All participants self-reported a clinical diagnosis of ASD (*N* = 24 ASD, 34 Asperger syndrome, 8 high-functioning autism, 4 autistic disorder, 1 PDD-NOS). The abridged version of the Autism Spectrum Quotient (AQ-28) [36] was used to confirm the autism characteristics. All participants exceeded the suggested AQ-28 score cut-off of >65 which has a sensitivity of .97 and a specificity of .82 for ASD. See Table 1 for descriptives.

**Table 1:** Descriptive statistics

	Mean (SD)	Sample range	Possible range	Shapiro-Wilk test (sig)
Chronological age	18.71 (2.51)	14.42–24.81	NA	*p* = .006
AQ-28	77.94 (8.93)	67–104	28–112	*p* = .001
Anxiety	12.90 (8.01)	0–31	0–36	*p* = .015
IS	2.17 (.58)	1.13–3.75	1–3	*p* = .34
Effortful control	68.11 (16.65)	33–103	19–152	*p* = .78

Note: IS score is calculated as mean (see Barrett et al. [37] for detail)

*NA* not applicable

### Procedures and measures

The results presented here form part of the nationwide Longitudinal Study of Australian Schools Leavers with ASD and their families within the Cooperative Research Centre for Living with Autism (Autism CRC) which was approved by the La Trobe University Human Ethics Committee and other institutional or organisational ethics committees as appropriate.

*Adult Repetitive Behaviours Questionnaire-2* (RBQ-2A) [37] is a self-report version of the original RBQ-2 [4] designed to measure a range of repetitive behaviours. The RBQ-2A has a stable two-factor structure with repetitive sensory-motor (RSM) and IS factors consistently identified across both normative development and in ASD [37].

*Effortful Control Domain of the Adolescent*/*Adult Temperament Questionnaire* [38] was used as a measure of self-regulation. It consists of 19 items assessing individual differences in the ability to perform actions when there is a strong tendency to avoid it, to focus and shift attention and to supress inappropriate approach behaviours. It is particularly geared toward attentional (example item: “When I am trying to focus my attention, I am easily distracted”) and inhibitory (example item: “It is easy for me to inhibit fun behaviour that would be inappropriate”) aspects. Higher scores indicate better EC capacity.

*DSM-5 Dimensional Anxiety Scales* (DSM-5 DAS) [39–41] is a brief screening questionnaire designed to provide both dimensional assessment of anxiety symptoms, as well as a threshold score (14) identifying clinically significant anxiety [41].

## Results

Internal consistency analysis (Cronbach’s *α*) for IS, anxiety and EC scales were 78, .92 and .84, respectively. The cut-off score for clinically significant anxiety was met by 40.6% of participants. All of the analyses were conducted using bootstrapping with 1000 resamples in order to generate more reliable, robust statistics. Females had significantly higher anxiety (17.12 [SD = 8.73] vs 10.93 [SD = 6.90], *F* = 10.15, *p* = .002, Cohen’s *d* = .79) and IS (2.41 [SD = .57] vs 2.07 [SD *=* .56], *F* = 5.39, *p* = .023, Cohen’s *d* = .60) scores. Although males had higher EC scores (69.31 [SD = 17.97] vs 66.32 [SD = 13.53]), this difference did not reach statistical significance *F* = .48, *p* = .49, Cohen’s *d* = .19.

Preliminary analysis showed no statistically significant association between chronological age and IS behaviours, anxiety, or effortful control scores. IS was associated with both EC (Pearson correlation coefficient [*r*] = −.39, *p* = .001, BCa 95% CI [−.55; −.20]) and anxiety (*r* = .45, *p* < .001, BCa 95% CI [24; .63]), and anxiety was in turn associated with EC (*r* = −.44, *p* < .001, BCa 95% CI [−.60; −.27]). There were no statistically significant differences between males and females in terms of direction and strength of correlations between anxiety, IS and EC (anxiety-IS correlations comparison: Fisher’s *z* = .66, *p* = 25; IS-EC correlations comparison: *z* = −.31, *p* = .39; anxiety-EC comparison: *z* = −1.88, *p* = .06). Therefore, the subsequent analyses were performed on the whole sample. In order to characterise this interrelationship, two mediation analyses were performed using the serial mediation model in PROCESS. PROCESS is a computational tool for mediation, moderation and mediated moderation that is run under Statistical Package for Social Science software (SPSS; version 21.0; [42]). The Bias-Corrected Accelerated Bootstrapped 95 percentile Confidence Intervals (BCa 95CI) with 5000 resamples was used as an inferential test for indirect effects in mediation analysis [43]. Indirect effects where BCa 95CI do not span zero are deemed as significant. Bias-Corrected Accelerated Bootstrapping method has advantages over the classic Baron and Kenny’s causal steps logic [44] as it does not require direct effect to be significant for a mediation to occur, and unlike Sobel test [45], it does not require data to be normally distributed [43]. Finally, it adjusts for measurement error when indirect effect is interpreted [46].

**Fig. 1:**
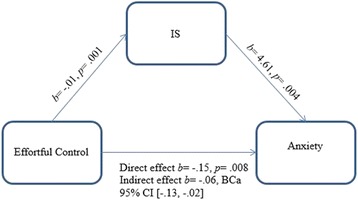
Mediation model 1

The first mediation model exploring whether the relationship between effortful control and anxiety was mediated by IS (see Fig. 1) was supported. A significant indirect effect of effortful control on anxiety, through IS, was found, *b* = −.06; BCa 95% CI [−.13, −.02]. The mediator accounted for 29.7% of the total effect (percent mediation [*Pm*] = .29, BCa 95% CI [.10, .65]).

**Fig. 2:**
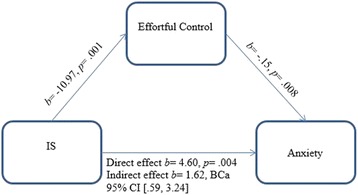
Mediation model 2

The second mediation model (see Fig. 2) exploring whether the relationship between IS behaviours and anxiety was mediated by effortful control was also supported. IS had an indirect effect on anxiety through effortful control, *b* = 1.62; BCa 95% CI [.59, 3.24]. The mediator accounted for approximately 26.04% of the total effect (*Pm* = .26, BCa 95% CI [.08, .58]).

## Discussion

Consistent with our predictions and previous literature [3, 10, 14, 25, 29, 34, 35], our study indicates that IS is positively associated with anxiety and that higher levels of anxiety and IS are both associated with lower levels of EC. Furthermore, our mediation analyses showed that the association between EC and anxiety was mediated by IS and that, in turn, the relationship between IS behaviours and anxiety was mediated by EC. Additionally, 40.6% of adolescents and young adults from our sample met the DSM-5 DAS criterion for clinically significant anxiety, which is in line with systematic reviews and larger studies [47–49] that suggest 40% as the most realistic prevalence figure for a clinical anxiety disorder.

IS behaviours have been implicated as playing similar roles in normative development and in neurodevelopmental and neuropsychiatric disorders by acting as a means of avoidance and thus reducing anxiety [14, 50]. Whereas in normative development, IS behaviours are gradually replaced by more mature and flexible forms of self-regulation that effectively regulate distress and anxiety, in ASD, IS behaviours persist as the primary means of self-regulation. Consequently, the inflexibility of IS behaviours will likely reinforce anxiety in the long term [34, 51]. Elevated and persistent IS behaviours can therefore impede the emergence of more developmentally appropriate modes of self-regulation in ASD, either because of the development of positive beliefs about the utility of IS [34, 51] or due to reduced exposure to situations that are conducive to developing more sophisticated self-regulatory strategies [10].

Using EC as a measure of self-regulation, our findings provide the first effort to disentangle the complex nature of the IS-anxiety-self regulation in the context of ASD. The mediation models offer support for both the developmental scenarios described above. Importantly, IS as mediator between EC and anxiety accounted for more variance than EC when mediating between IS and anxiety. These findings are nevertheless limited by the cross-sectional design, relatively older age of the sample, making the directionality of relationships difficult to establish. Cross-sectional designs are unable to capture these dynamic processes as they develop and unfold over time, and it is clear that a carefully designed longitudinal study, with a larger, well-characterised sample and multi-method assessments to avoid potential bias due to the common method variance, is necessary. In addition, due to the gender differences in anxiety and IS present in both typically developing and ASD populations [5, 52, 53], it will be important for the future research to address potential differences in underlying mechanisms between males and females. The sample size used here was in line with other studies of this type, and the application of the Bias-Corrected Accelerated Bootstrapped 95th percentile confidence intervals test as a method has been shown to be more robust in studies with sample sizes such as ours [46] in order to test indirect effect. However, replication in a larger sample is needed. Therefore, although an important first step, our results should be considered as preliminary at this point. Nevertheless, they have potential implications for interventions targeting IS and anxiety in ASD.

Interventions specifically focused on RRBs in children with ASD and other neurodevelopmental disorders are rare [54, 55]. Our findings suggest self-regulation as a viable intervention target as measured by EC. A number of interventions targeting both EC and EF, for example Tools of Mind [56], have been shown to increase executive attention in children aged 3–7 years [57]. Similar interventions have been shown to be effective in improving EF in children with attention-deficit hyperactivity disorder [58]. In addition, Unstuck on Target [59], a behavioural approach to develop flexibility and compensatory strategies for impairments in EF, has been shown to increase problem-solving, flexibility, and planning/organizing aspects of executive functioning based on experimental assessments and parental reports, as well as enabling easier transitions and improving flexibility within classrooms for children with ASD [60]. Therefore, it will be important in future work to explore the effects of these types of interventions on IS and anxiety.

## Conclusions

Our study explored self-regulation and its relationship with IS and anxiety from a temperament perspective using a measure of EC. In ASD research, self-regulation has been mainly considered from an EF framework, and indeed, impairments in EF have been linked with repetitive behaviours, although with mixed results [5], as well as with anxiety [29, 31]. Although the majority of research suggests EC and EF to be largely overlapping constructs [17, 18], future research should consider self-regulation using measures from different theoretical frameworks, integrating measures of EC and EF, as well as a process model of emotion regulation [61]. Future progress in this area will rely on the study of self-regulation using measures of both EC and EF, as well as of emotion regulation, taken from multiple sources and from multiple informants, together with experimental/laboratory tasks such as the Attention Network Test (ATN) [62] and intervention designs.

## Abbreviations

AQ: Autism Spectrum Quotient
ASD: Autism spectrum disorder
Autism CRC: Cooperative Research Centre for Living with Autism
EC: Effortful control
EF: Executive function(s)
IS: Insistence on sameness
